# Information diversity and health care quality: The role of trust in health care system and self-care confidence

**DOI:** 10.1093/geroni/igaf122.4395

**Published:** 2025-12-31

**Authors:** Jeong Eun Lee, In Jeong Hwang, Ansuk Jeong, Yulri Kim

**Affiliations:** Iowa State University, Ames, Iowa, United States; Iowa State University, Ames, Iowa, United States; DePaul University, Chicago, Illinois, United States; Iowa State University, Iowa State University, Iowa, United States

## Abstract

With health information now accessible through diverse channels, older adults are increasingly seeking it from multiple sources using online portals, social media and wearable devices. Whether these emerging health-seeking behaviors complement or conflict with traditional practices such as visiting doctors warrants an empirical investigation. This study first examines the relationship between information-seeking behavior and perceived quality of professional healthcare services and then explores whether this relationship is moderated by trust in the healthcare system and confidence in self-care. Based on 2,203 older adults over 65 (M = 73.3, SD = 6.7) from wave 6 of HINTS (Health Information National Trends Survey), we create health information diversity scores based on standardized measures of health information frequency and variety—derived from patient engagement with online portals, social media, and wearable devices. We then analyze how these diversity scores relate to perceived care quality from health professionals, with healthcare system trust and self-care confidence as moderators. We found that diverse information was in general negatively associated with perceived care quality. However, both healthcare trust and self-care confidence moderated this relationship. In particular, the negative relationship weakened with high trust and hjgher self-care confidence compared to those with lower levels. Health information source diversity may undermine care satisfaction from health care professionals, especially when patients lack trust in healthcare system or confidence in self-management. Healthcare interventions should prioritize building both institutional trust and patient self-efficacy to help patients effectively navigate diverse health information while maintaining positive care experiences.

